# ATOH1 Can Regulate the Tumorigenicity of Gastric Cancer Cells by Inducing the Differentiation of Cancer Stem Cells

**DOI:** 10.1371/journal.pone.0126085

**Published:** 2015-05-07

**Authors:** Myoung-Eun Han, Su-Jin Baek, Seon-Young Kim, Chi-Dug Kang, Sae-Ock Oh

**Affiliations:** 1 Department of Anatomy, School of Medicine, Pusan National University, Pusan, Republic of Korea; 2 Department of Biochemistry, School of Medicine, Pusan National University, Pusan, Republic of Korea; 3 Medical Research Center for Ischemic Tissue Regeneration, Pusan National University, Pusan, Republic of Korea; 4 Medical Genomics Research Center, KRIBB, Daejeon, Republic of Korea; Second University of Naples, ITALY

## Abstract

Cancer stem cells (CSCs) have been shown to mediate tumorigenicity, chemo-resistance, radio-resistance and metastasis, which suggest they be considered therapeutic targets. Because their differentiated daughter cells are no longer tumorigenic, to induce the differentiation of CSCs can be one of strategies which can eradicate CSCs. Here we show that ATOH1 can induce the differentiation of gastric cancer stem cells (GCSCs). Real time PCR and western blot analysis showed that ATOH1 was induced during the differentiation of GCSCs. Furthermore, the lentivirus-induced overexpression of ATOH1 in GCSCs and in gastric cancer cell lines significantly induced differentiation, reduced proliferation and sphere formation, and reduced *in vivo* tumor formation in the subcutaneous injection and liver metastasis xenograft models. These results suggest ATOH1 be considered for the development of a differentiation therapy for gastric cancer.

## Introduction

After cancer stem cells (CSCs) were initially isolated from leukemia patients, they had been isolated from many cancer types, including gastric cancer [1,2,3,4,5,6]. CSCs have been suggested to be a therapeutic target because they can mediate tumorigenicity, chemo-resistance, radio-resistance and metastasis [7,8,9]. Conventional anti-cancer therapy mainly targets proliferating non-CSCs, which explains the frequent recurrence of cancer [9]. The poor prognosis of patients with greater CSC population, also encourages the development of therapeutics that target CSCs [10].

One of characteristics of CSCs is that they can differentiate into daughter cells which are no longer tumorigenic, for example, CSCs from colon and stomach cancers have been induced to differentiate by serum [5,6]. This means the CSC hypothesis differs fundamentally from the traditional clonal expansion hypothesis. Furthermore, the hierarchical relationship between CSCs and their daughter cells can be explained by epigenetic mechanisms that can be applied to normal stem cells [11,12]. In addition, the microenvironment can affect the differentiation of CSCs [13], and this characteristics can be exploited for the development of therapeutics.

Accordingly, the induction of differentiation is a strategy that can be used to eradicate CSCs. Epigenetic regulators have been suggested to induce their differentiation, for example, histone deacetylase (HDAC) inhibitors have been shown to induce differentiation of CSCs in leukemia which were caused by fusion proteins, such as, AML1-ETO and PML-RARα [14,15]. Furthermore, all-trans retinoic acid (ATRA) can induce differentiation of CSCs in acute promyelocytic leukemia (APL) via C/EBP factors [16,17], wild type transcription factors, such as, C/EBPα, can induced differentiation in human AML and in lung cancer cell lines and in mouse *in vivo* models [17,18,19,20]. In gastric cancer, suramin has been shown to induce differentiation of human gastric cancer cell lines [21].

ATOH1 is a basic helix-loop-helix (bHLH) transcription factor homologous to the Drosophila atonal[22]. It activates E box-dependent transcription in collaboration with E47[23]. HES1, a molecule in Notch signaling pathway, can inhibit its expression[24]. Knock-out mouse studies have shown that ATOH1 plays critical roles in the generation of cerebellar granule neurons, inner ear hair cells, spinal cord interneurons, Merkel cells in the skin and intestinal secretory cells[25]. Interestingly, ATOH1 has been associated with various kinds of cancers including colon cancer[25].

To development of a new differentiation therapy in gastric cancer, we analyzed expression changes at the mRNA levels during the differentiation of gastric cancer stem cells (GCSCs). It was found that during the differentiation of GCSCs, the expression level of ATOH1, which is important for the differentiation of intestinal epithelial cells [26,27], was significantly increased. In addition, the induction of ATOH1 in gastric cancer cell lines and in GCSCs significantly reduced their tumorigenicity in subcutaneous injection and liver metastasis models.

## Materials & Methods

### Gastric cancer stem cell cultures

Gastric cancer tissues were retrieved after obtaining written informed consent from patients that underwent surgical resection at Pusan National University Hospital (PNUH) and Pusan National University Yangsan Hospital (PNUYH). The study protocol was approved by Pusan National University Hospital-Institutional Review Board (PNUH-IRB) and Pusan National University Yangsan Hospital-Institutional Review Board (PNUYH-IRB). Gastric cancer stem cells were cultured as previously described [6], and were induced to differentiate by adding 5% FCS to media instead of growth factors.

### Culture of gastric cancer cell lines

Gastric cancer cells (SNU1, SNU16, SNU216, SNU620, SNU638, NCI-N87) were cultured in RPMI1640 supplemented with 10% fetal bovine serum (FBS) and 100 *μg*/ml of penicillin/streptomycin at 37°C in a 5% CO_2_ humidified incubator. These cell lines were obtained from Korean Cell Line Bank (Seoul, Korea).

### Culture of 293FT cell line

293FT cells were maintained in DMEM supplemented with 10% fetal bovine serum and 0.1 mM MEM Non-Essential Amino Acids (NEAA), 1 mM MEM sodium pyruvate (both from SIGMA, Sigma-Aldrich, St. Louis, MO, USA), 6 mM L-glutamine (Life Technologies, Grand Island, NY, USA), and 1% Pen-Strep in a 5% CO_2_ humidified incubator.

### RNA sequencing and data analysis

Library was prepared using the IlluminaTruSeq RNA Sample Prep Kit and sequenced using the Illumina Genome Analyzer IIx (San Diego, CA, USA) to generate 76 bp paired end reads. Sequenced reads were aligned on a human reference genome 19 using Tophat 2 [pubmed id 19289445]. mRNA expression was measured as RPKM (Reads Per Kilobase per Million mapped reads) from uniquely mapped reads using a hg19 RefSeq model and the HTSeq package (http://www-huber.embl.de/users/anders/HTSeq/). Bed files were produced using Tophat 2. All RNA sequencing data were deposited at the public repository (GEO Accession Number: GSE46597).

### Real time PCR

Extraction of RNA and real time PCR were carried out using a Power SYBR Green PCR Master Mix and an ABI Prism 7500 sequence detector (both from Applied Biosystems, Foster City, CA, USA), as previously described [28]. The primer sequences used (forward and reverse, respectively) were as follows: Differentiation markers; CA1, 5’- TCA GAG CAG CTG GCA CAA TTC CG -3’ and 5’- CCT TCA GAG GTT GGG TTG GGC G -3’; SSTR2, 5’- CCA GGA ACC CCA AAC GTC CG -3’ and 5’- CAG TGT GAC ATC TTT GCT TTT CCG C -3’; CHGA, 5’- GCA AAC CGC AGA CCA GAG GAC C -3’ and 5’- TGT CTC AGC CCC GCC GTA GT -3’; PGC2, 5’- TGG CCT ACC CTG CTC TGT CCG -3’ and 5’- ACT CCA GGA CCA GGT TGC TGA GG -3’; MUC5AC, 5’- TCA CAC GGT CCT GCC CAC AGA G -3’ and 5’- ACC CCT GCA TCT CAG AGC CCC -3’; ATP4B, 5’- GAA AGC CCA GCC CCA CTA CAG C -3’ and 5’- TGC TCC GCC ATG ACC TTG CAC -3’; Stemness markers; CD44, 5’- GCC TGG GGA CTC TGC CTC GT -3’ and 5’- AGC CTT GCA GAG GTC AGC GG -3’; LGR5, 5’- GAG CTG CCT TCC AAC CTC AG -3’ and 5’- CCC GCA AGA CGT AAC TCC TC -3’; BMI1, 5’- TGG TTG CCC ATT GAC AGC GG -3’ and 5’- AAA AAT CCC GGA AAG AGC AGC CG -3’; and c-Myc, 5’- ACA CCC GAG CAA GGA CGC GA -3’ and 5’- CGC GGG AGG CTG CTG GTT TTC -3’; ATOH1, 5’- CCC CTT CCA GCA AAC AGG TG -3’ and 5’- ACG GGA TAA CAT TGC GCA GC -3’; GAPDH 5’- GAG TCC ACT GGC GTC TTC AC -3’ and 5’- ATG ACG AAC ATG GGG GCA TC -3’. GAPDH was used to standardize cDNA input levels.

### Western blot analysis

Western blot analysis were performed as previously described [24], using the following antibodies: ATOH1 (LSBio, Seattle, WA, USA); CD44, LGR5, ATP4B, CA1, PGC, MUC5AC (Sigma-Aldrich Co., St. Louis, MO, USA); c-Myc, caspase-3 (Santa Cruz Biotechnology, CA, USA); cleaved caspase-3, caspase-9, cleaved caspase-9 (Cell Signaling Technology, MA, USA); p21, β-actin antibody (Abcam, Cambridge, MA, USA) and HRP-conjugated secondary antibody (Jackson ImmunoResearch Laboratories, West Grove, PA, USA).

### Lentiviral constructs and the generation of active virus

To create an expression clone, sequence verified viral ORF Clones (ATOH1, IOH34744; Life Technologies, Grand Island, NY) in Gateway entry vectors were transferred into pLenti7.3-DEST vector (pLenti7.3⁄V5-DEST Gateway Vector Kit, Life technologies, Grand Island, NY, USA) using an LR recombination reaction (LR Clonase II enzyme mix, Life technologies, Grand Island, NY, USA). For transformation, One Shot Stbl3 Competent E. coli (Life Technologies) was used. After cotransfection of the expression clone and the ViraPower Packaging Mix (Life Technologies) into the 293FT Cell Line (Life Technologies) to produce lentivirus, lentiviral supernatants were harvested. These lentiviral stocks were used to transduce GCSCs and gastric cancer cell lines.

Virus collection and propagation were performed according to the manufacturer's instructions (Life Technologies). The control virus was generated in parallel without the inclusion of ATOH1.

### Construction of RASSF4-reporter cell lines and luciferase assay

The Gluc-ON Promoter Reporter Clones, pEZX-PG02 (HPRM11834-PG02, GeneCopoeia, Rockville, MD, USA) were used to construct RASSF4-luciferase reporter cell lines (NCI-N87 and SNU216 cells) and puromycin (Sigma-Aldrich, St. Louis, MO, USA) was used for selection. The lentivirus described above was used to induce ATOH1. Luciferase activities were measured using Secrete-Pair Gaussia Luciferase Assay Kit (GeneCopoeia, Rockville, MD, USA) according to manufacturer’s instructions at 72 h post-transduction.

### Xenograft assay

Cells were diluted to an appropriate injection dose (1x10^6^ cells), mixed with BD Matrigel (BD Biosciences, CA, USA) at a 1:1 ratio, and injected subcutaneously on the dorsal side of each flank in Severe combined immunodeficiency (SCID) mice (n = 5/group). To minimize experimental variability due to individual differences in recipient mice, cell populations that were to be compared were injected on opposite flanks of the same animals. Tumors were harvested after 4 weeks and tumor weights were measured. For the liver metastasis model, SCID mice were anesthetized with an intra-peritoneal injection of Ketamine/Xylazine combination. Then the mice were incised about 10 mm on the left subcostal, the spleen was confirmed under the peritoneum, the peritoneum was opened for about 8mm and the spleen was exposed over the peritoneum. The cells (5x10^4^ cells/100 *μl*) were slowly injected into the spleen of mice via a 27-gauge needle (n = 5/group) and then the spleen was returned to the abdominal cavity, the peritoneum and the wound was sutured. 4 weeks after inoculation with cells, the mice were sacrificed [37]. This study was carried out in strict accordance with the recommendations in the Guide for the Care and Use of Laboratory Animals issued by the National Institute of Health. Pusan National University-Institutional Animal Care and Use Committee (PNU-IACUC) approved all experimental procedures involving animals.

### Cell proliferation assay

Five days following transduction with lentivirus, 10*μl* of pre-mixed water-soluble tetrazolium salt-1 (WST-1, Roche, Indianapolis, IN, USA) was added into wells. These cells were then incubated for two hours and cell viability was determined by measuring absorbance at 450 nm using an ELISA reader (TECAN, Mannedorf, Switzerland).

### Sphere formation assay

Spheres were dissociated into single cells and plated in 96-well plates in 0.2 ml volumes of media. Each well of the 96-well plate contained less than 10 cells. Cells were then cultured and monitored for 5–7 days. Spheres larger than 25 μm were counted using the inverted microscope and the efficiency of sphere formation was compared.

### Data analysis

The nonparametric Mann-Whitney U-test or the Student’s t-test (unpaired comparisons) were used to determine the significances of differences between the mean values of two groups, and one-way analysis of variance (ANOVA) followed by Tukey’s multiple comparisons was used to determine the significances of differences between the mean values of three or more groups. * indicates a P value of <0.05, which was regarded the significance criterion. Data were analyzed using SPSS software, version 12.0 (SPSS Inc., Chicago, IL, USA). Results are presented as means±SDs.

## Results

### Induction of ATOH1 during the differentiation of GCSCs

**Fig 1:**
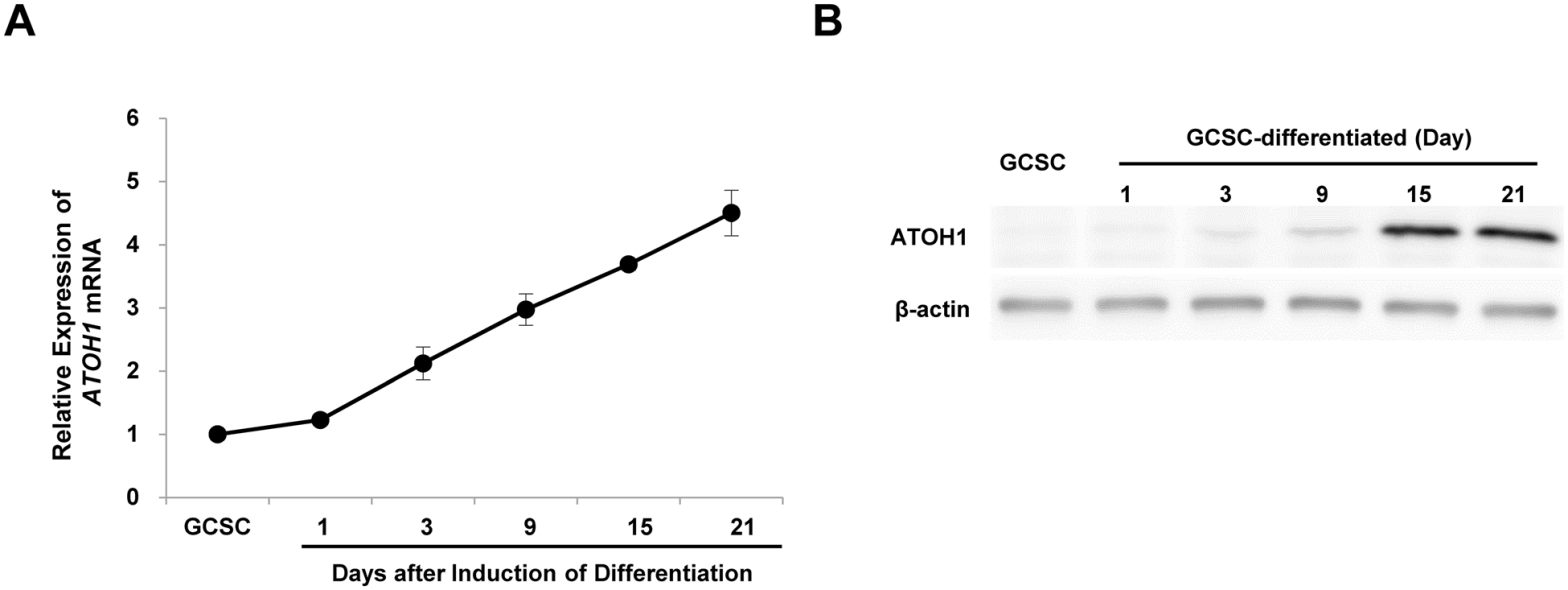
Induction of ATOH1 during the differentiation of GCSCs. The differentiation of GCSCs was induced by adding FBS. mRNA expression levels were determined by real time PCR (A). Results are expressed as the means ±SDs of three independent experiments. Protein expression levels were determined by western blot analysis (B).

The differentiation of GCSCs by serum has been previously demonstrated [6]. To identify factors associated with differentiation, we compared the mRNA expression profiles of stem cells and cells in differentiated cell states by RNA sequencing. Many tumor suppressor genes were induced during the differentiation (data not shown). Interestingly, ATOH1, a transcription factor critical for the differentiation of intestinal epithelial cells, was also induced. To confirm this induction of ATOH1 during differentiation, we examined changes in the expression level of ATOH1 by real time PCR and western blot analysis. We found as GCSCs approached terminal stages of differentiation, the expression of ATOH1 increased (Fig 1).

### Overexpression of ATOH1 induced the differentiation of GCSCs

**Fig 2:**
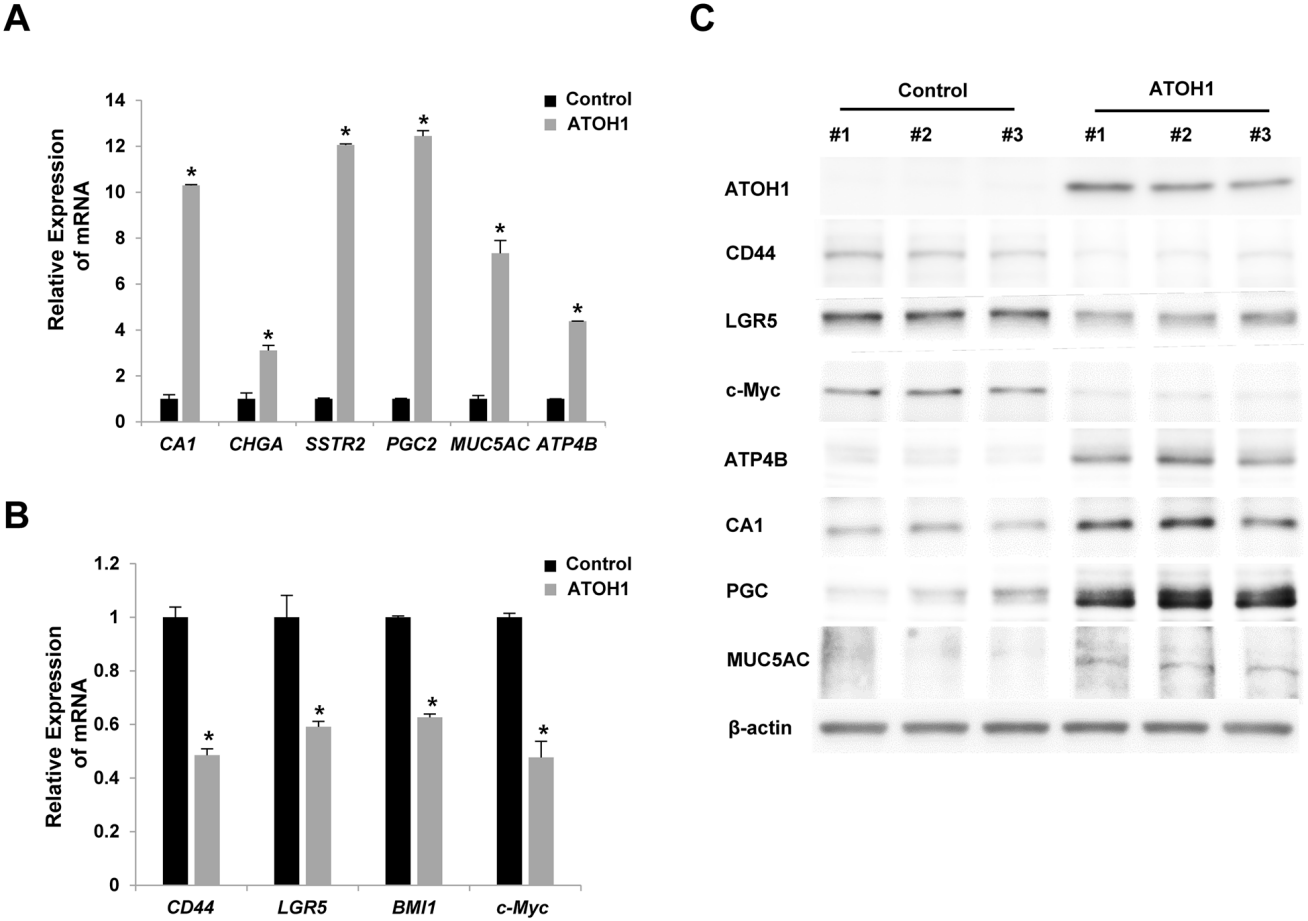
Overexpression of ATOH1 induced the differentiation of GCSCs. Various markers for differentiation (A) or stemness (B) were examined after overexpressing ATOH1 in GCSCs by real-time PCR (A, B) and western blot analysis (C). Results are expressed as the means ±SDs of three independent experiments. * *P* < 0.01.

To determine the roles of ATOH1 during GCSC differentiation, we overexpressed it in GCSCs using lentivirus. To check differentiation statuses, we examined the expression levels of various markers for differentiation or stemness. Notably, the overexpression of ATOH1 in GCSCs increased the expression level of various differentiation markers, but decreased the expression levels of various stemness markers (Fig 2).

**Fig 3:**
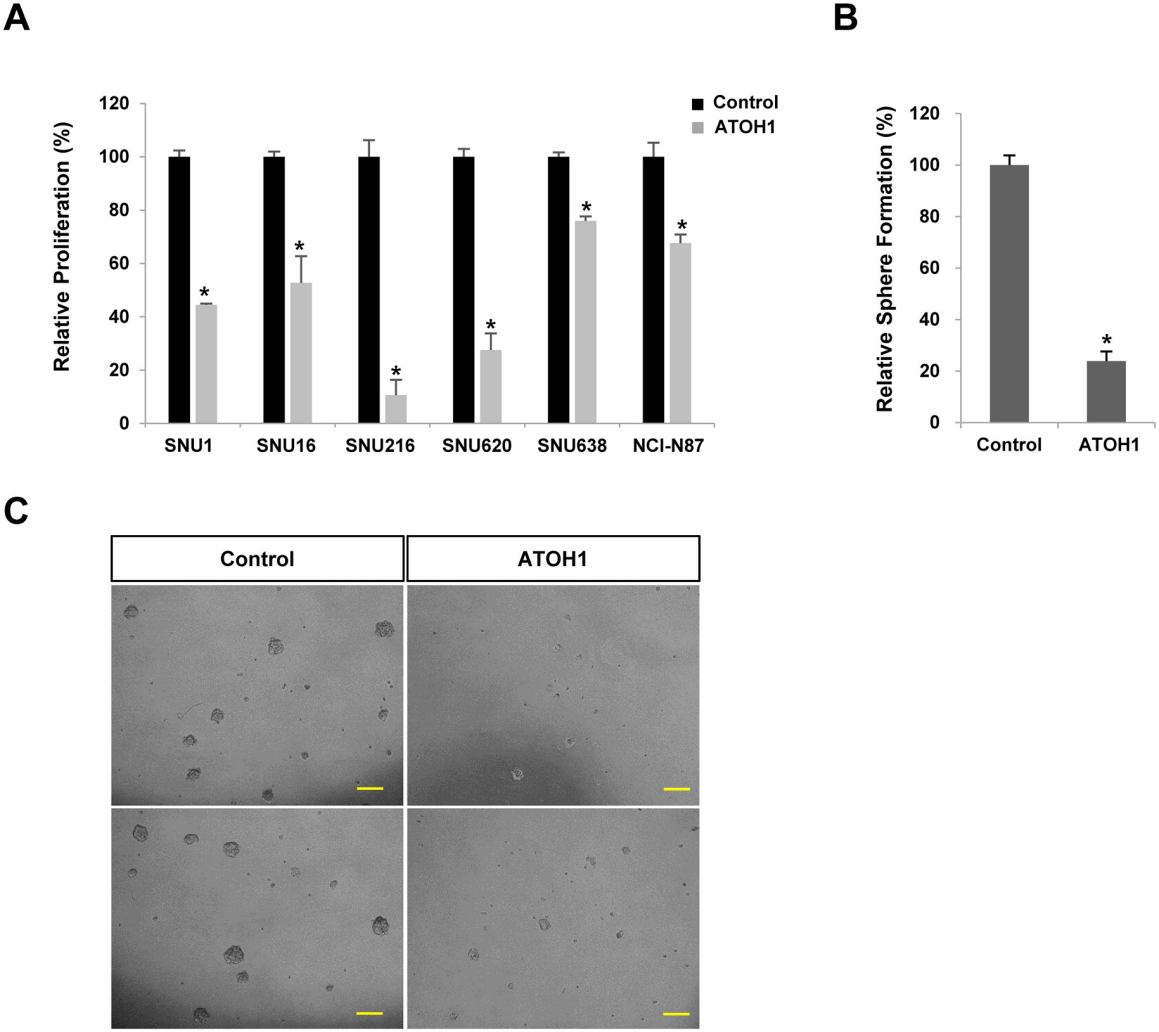
Overexpression of ATOH1 reduced the proliferation of various gastric cancer cell lines (A) and reduced sphere formation by GCSCs (B-C). Scale bar = 100*μm*. Results are expressed as the means ±SDs of three independent experiments * *P* < 0.01.

Next we investigated whether ATOH1 could regulate proliferation or sphere formation. Interestingly, the overexpression of ATOH1 significantly decreased the proliferations of various types of gastric cancer cell lines and sphere formation by GCSCs (Fig 3).

### ATOH1 overexpression reduced *in vivo* tumorigenicity

**Fig 4:**
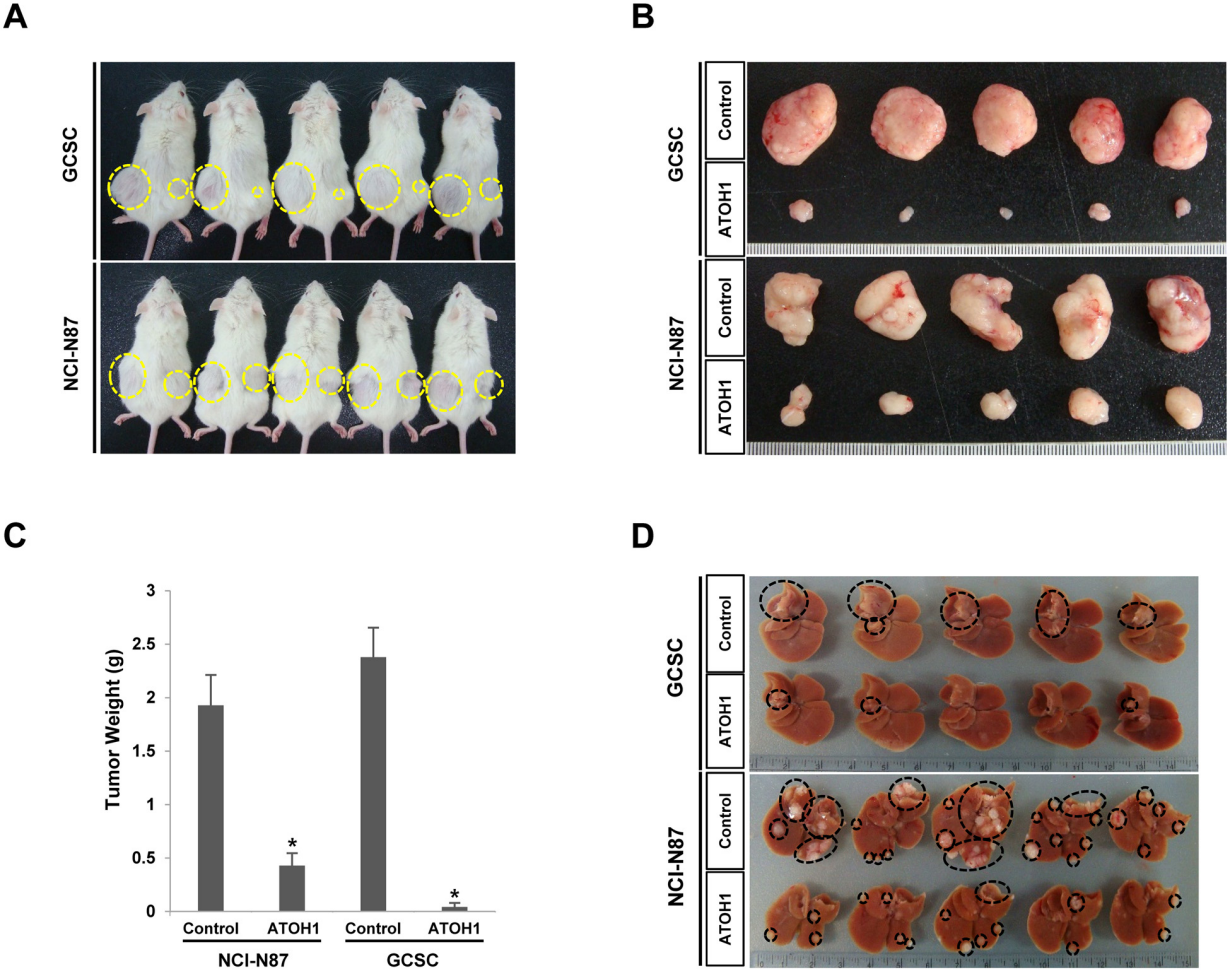
Overexpression of ATOH1 reduced the *in vivo* tumorigenicity of GCSCs and of NCI-N87 gastric cancer cells in the subcutaneous injection (A, B, C) and liver metastasis model (D). After 4 weeks, the tumor masses were obtained and their weights measured (C).

To determine whether ATOH1 can regulate the *in vivo* tumorigenicity of gastric cancer cells, we used a liver metastasis model produced by injecting gastric cancer cells into the spleen (cells subsequently migrated to liver). Notably, the overexpression of ATOH1 significantly reduced tumor formation in liver by GCSCs and by NCI-N87 gastric cancer cells (Fig 4). Moreover, when we made subcutaneous xenografts, similar results were observed (Fig 4).

### ATOH1 increased RASSF4 promoter activity

**Fig 5:**
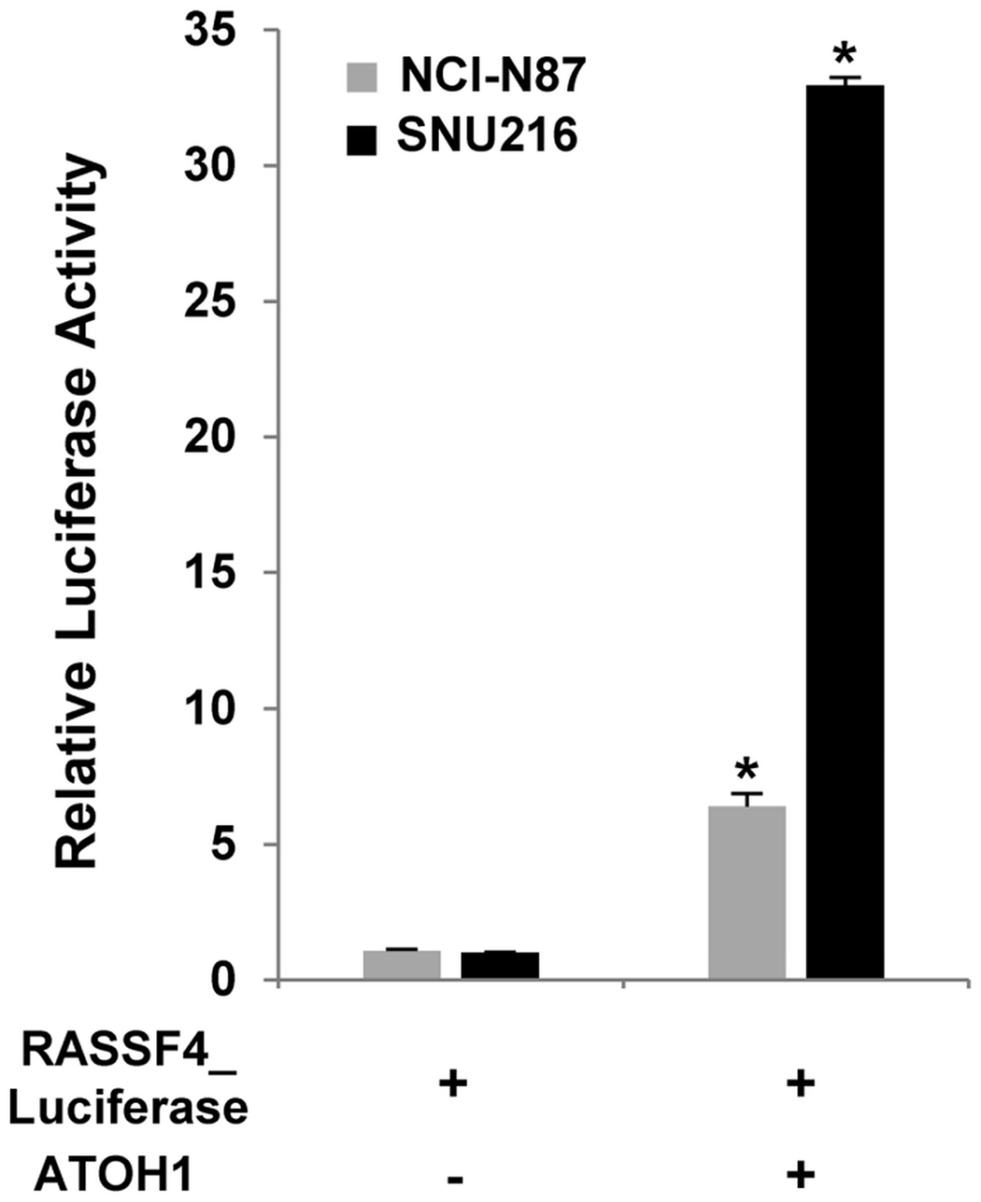
ATOH1 increased RASSF4 promoter activity. The RASSF4 promoter coupled with the luciferase gene was introduced into NCI-N87 and SNU216 cells. Transduction with ATOH1 significantly increased the luciferase activity. Data are the means±SD of three independent experiments * *P* < 0.01.

To confirm whether the differentiation of GCSCs by ATOH1 is mediated through its transcriptional activity, we examined the promoter activity of an ATOH1 target gene (RASSF4) using luciferase assays[29]. After the RASSF4 promoter, which was coupled with the lucifease gene, was stably introduced into the gastric cancer cell lines (NCI-N87 and SNU216 cells), we examined effects of ATOH1 on the activity of luciferase. Transduction with ATOH1 significantly increased the luciferase activity of RASSF4 promoter. (Fig 5).

### The involvement of apoptosis and cell cycle pathways in the effects of ATOH1

Previous reports have reported apoptosis and cell cycle pathways are involved in the effects of ATOH1 on tumors in retina[30] and colon[25]. So, we examined whether related proteins are also involved in the effects of ATOH1 on proliferation (Fig 3A) and sphere formation (Fig 3B) in the present study. The change in proliferation and sphere formation could be due to a slower cell cycle, an increased apoptotic rate, or both.

**Fig 6:**
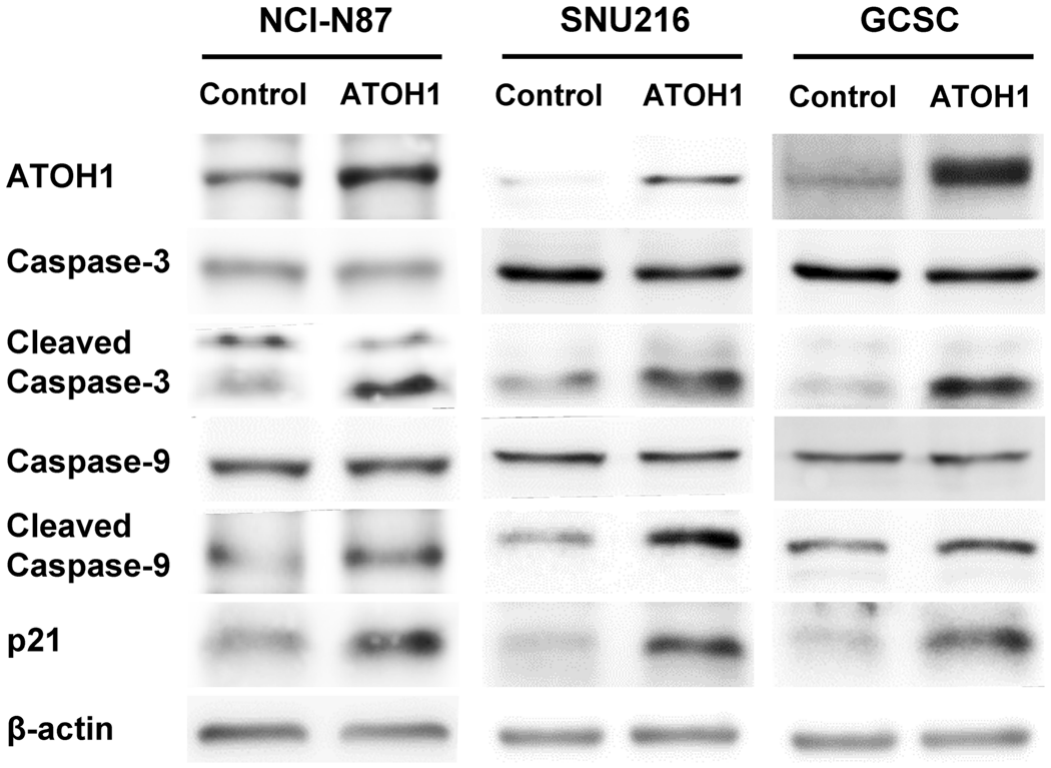
Activation of caspases and induction of p21 by ATOH1. Total and cleaved forms of caspase 3 and 9 were examined 2 days after the transduction with ATOH1 in NCI-N87 cells, SNU216 cells, and GCSCs. Cleavage of caspase 3 and 9, up-regulation of p21 were observed in ATOH1-overexpressed cells.

We found the transduction with ATOH1 increased the level of cleaved-caspase-3 and—caspase-9 in GCSCs and gastric cancer cell lines (Fig 6), suggesting that the intrinsic apoptosis pathway might be involved. Moreover, we also found up-regulation of p21 upon ATOH1 overexpression (Fig 6).

## Discussion

In the present study, we show that ATOH1, a HLH transcription factor, can induce the differentiation of GCSCs, and this leads to a loss of tumorigenicity. ATOH1 overexpression has been previously shown to induce the differentiation of intestinal stem cells into secretory cells [24], and in ATOH1-null mice, secretory cells were not generated in intestine [26]. This effect of ATOH1 on differentiation has also been observed in colorectal cancer cells, in which it acted as a tumor suppressor. Furthermore, in approximately 70% of colorectal cancers, its expression has been reported to be diminished by genetic and epigenetic mechanisms [25,31]. In colorectal cancer cells, ATOH1 overexpression reduced proliferation and growth in soft agar and xenografts [31], and deletion of ATOH1 enhanced tumorigenicity in mice [25]. These results collectively suggest that ATOH1 can be utilized as a differentiation therapy for gastrointestinal cancers.

In contrast with its effect on differentiation, ATOH1 can also promote the proliferation of progenitor cells and tumor formation. During cerebellar development of mice, ATOH1 was found to initiate the differentiation program of cerebellar granule neuron precursors at an early stage (P0). However, at a later stage (P5), it promoted the proliferation of precursors [32,33,34]. ATOH1 deletion at P0, strongly inhibited differentiation, but its deletion at later stage resulted in exit from the cell cycle and differentiation [35]. These different roles of ATOH1 at different stages can be explained by different targetomes [35]. ATOH1 promoted medulloblastoma formation together with the Shh hedgehog signaling pathway [36,37]. Furthermore, its expression was found to be significantly elevated in mucinous adenocarcinoma, signet ring carcinoma, and intestinal neuroendocrine tumors [38]. Therefore, to be able to utilize ATOH1 for differentiation therapy, its direct targets that induce differentiation need to be identified.

Although the expressional statuses of ATOH1 have been reported in normal gastric mucosa and in gastric cancer, its roles are poorly characterized. ATOH1 is expressed in normal human gastric epithelial cells at low levels [39,40], but its expression was not observed in normal gastric epithelial cells in the mouse stomach [26]. Furthermore, mouse and human metaplastic gastric mucosa showed ATOH1 expression [41], but its expression was not observed in gastric cancer cell lines, including MKN74, MKN45, KATOIII, and HSC58 [40]. In some gastric cancer patients, it is overexpressed [39]. Its pathophysiological roles have never been explored in gastric cancer. In the present study, we found that the induction of ATOH1 expression can reduce the proliferation, invasion, and tumorigenicity of gastric cancer cells. We also found the possible involvement of p21 and apoptosis pathway in ATOH1’s effects.

Our observations suggest the possibility that the ATOH1 gene should be viewed as potential treatment target for gastric cancer. Small molecules that induce its expression or gene therapy using virus or stem cells could be developed. In particular, ATOH1 was found to induce the differentiation of gastric cancer stem cells, and thus, therapeutics targeting ATOH1 might be able to eradicate cancer stem cells.
